# Starch Chemical Composition and Molecular Structure in Relation to Physicochemical Characteristics and Resistant Starch Content of Four Thai Commercial Rice Cultivars Differing in Pasting Properties

**DOI:** 10.3390/polym15030574

**Published:** 2023-01-22

**Authors:** Wichian Sangwongchai, Kanitha Tananuwong, Kuakarun Krusong, Supidcha Natee, Maysaya Thitisaksakul

**Affiliations:** 1 Biological Science Program, Faculty of Science, Khon Kaen University, Khon Kaen 40002, Thailand; 2 Department of Food Technology, Faculty of Science, Chulalongkorn University, Bangkok 10330, Thailand; 3 Center of Excellence in Structural and Computational Biology, Department of Biochemistry, Faculty of Science, Chulalongkorn University, Bangkok 10330, Thailand; 4 Department of Biochemistry, Faculty of Science, Khon Kaen University, Khon Kaen 40002, Thailand; 5 Salt Tolerant Rice Research Group, Faculty of Science, Khon Kaen University, Khon Kaen 40002, Thailand

**Keywords:** rice (*Oryza sativa* L.), rice starch, resistant starch, starch molecular structure, starch physicochemical properties, starch functional properties

## Abstract

Variations in starch pasting properties, considered an alternative potential quality classification parameter for rice starches, are directly controlled by the diverse starch molecular composition and structural features. Here, the starch characteristics of four rice cultivars (i.e., RD57, RD29, KDML105, and RD6) differing in pasting properties were assessed, and their relationship was determined. The results revealed that protein and moisture contents and their crystalline type were similar among the four rice starches. However, their molecular compositions and structures (i.e., reducing sugar and amylose contents, amylopectin branch chain-length distributions, granule size and size distribution, and degree of crystallinity) significantly varied among different genotypes, which resulted in distinct swelling, solubility, gelatinization, retrogradation, and hydrolytic resistance properties. The swelling power and gelatinization enthalpy (∆H) were positively correlated with C-type granule and relative crystallinity, but were negatively correlated with amylose content, B-type granule and median particle size (d(0.5)). Conversely, the water solubility and resistant starch content negatively correlated with C-type granule, but positively correlated with amylose content, B-type granule, and d(0.5). The gelatinization onset temperature (To(g)), and retrogradation concluding temperatures (Tc(r)), enthalpy (∆H(r)), and percentage (R%) were positively impacted by the amount of protein, amylose, and B1 chains (DP 13–24), while they were negatively correlated with short A chains (DP 6–12). Collectively, the starch physicochemical and functional properties of these Thai rice starches are attributed to an interplay between compositional and structural features. These results provide decisive and crucial information on rice cultivars’ suitability for consumption as cooked rice and for specific industrial applications.

## 1. Introduction

Rice starch is one of the world’s most important sources of food energy [1]. In Thailand, rice grains are the major food and give the most caloric intake for 70.08 million individuals [2,3]. Currently, rice starches isolated from Thai rice grains are widely used as biopolymers in the beverages, bakery, pharmaceutical and supplement, cosmetic, animal feed, paper making, adhesive, and textile industries [4,5]. In 2021, starch-rich food and non-food products from Thai commercial rice cultivars were exported to the world market, providing about USD 4087 million in income for the country [6]. The extensive industrial applications of Thai commercial rice starches are due to their great variations in molecular compositions and structures (i.e., amylose and protein contents, amylose–lipid complex, chain length distribution (CLD) of amylopectin, average chain length (CL), degree of polymerization, granule size, and granule crystallinity) [2,5,7,8,9]. These are critical determinants for their diversities in non-resistant and resistant starch (RS) contents and physicochemical properties (e.g., swelling, solubility, thermal properties, and pasting properties) [2,8,10,11,12].

Starch chemical composition and molecular structure can affect starch physicochemical properties and in vitro digestibility, which ultimately determines the final starchy-food product quality. Due to the wide genotypic diversity, starches from various Thai rice cultivars have been the focus of increasing research interest [5,8,10]. Rice starch mainly consists of linear amylose and highly branched amylopectin, which are organized into a semi-crystalline structure of starch granules [13]. The semicrystalline growth rings of starch granules are constructed by the alternating amorphous and crystalline lamellae with a repetitive distance of 9–10 nm [13]. The amorphous lamella consists of loosely packed amylose chains and amylopectin branch points, whereas the crystalline lamella is formed by densely packed short- and long-double helices of amylopectin branch chains [13]. Consequently, the amylopectin double helices that are packed in an ordered structure primarily contribute to the formation of the crystallinity of starch granules [13]. Longer starch chains would form long or strong single and double helices through intra- and intermolecular hydrogen bonds between chains, spanning the complete crystalline structures [2,8,9,10,14,15]. In contrast, the presence of shorter starch chains would produce shorter or weaker single and double helices, resulting in inferior crystalline structures within the granules [2,8,9,10,14,15]. Therefore, the role of amylose content and amylopectin fine structure on starch physicochemical and functional characteristics and in vitro digestibility has been extensively studied [8,9,10,14,16]. Rice starches containing large amounts of amylose and amylopectin long branch chains (DP ≥ 37) displayed a high starch solubility, a low swelling power, and high gelatinization temperatures and gelatinization enthalpy (ΔH) due to the greater requirement of thermal energy to complete granular dissociation. They also had high pasting temperature (PT), setback (SB) and final viscosity (FV) values of starch gel, high RS content due to their resistance to hydrolysis by digestive enzymes, and low peak viscosity (PV) and breakdown (BD) values of starch paste [2,8,9,10,14,15]. The proportion of short A chains (DP 6–12) had a negative influence, while the amounts of amylose and longer amylopectin chains (DP 13–24) had a positive influence on the gelatinization temperatures, PT, SB, and RS content [8,10,16]. In addition, the granule relative crystallinity was positively correlated with swelling power and gelatinization temperatures (i.e., the onset (To(g)), peak (Tp(g)), and conclusion (Tc(g)) temperatures) and ΔH(g) of starch [14]. On the other hand, amylose content and starch granule size were negatively and positively correlated with PV value, respectively [8]. On the other hand, a few works have described the link between starch molecular structure and its digestibility [10]. Amylopectin CLD was previously shown to associate with the lamellar structures of starch granules, which directly influence the enzymatic accessibility and the enzyme hydrolysis rate of native and cooked rice starch during digestion [9,10,17]. The proportion of DP 13–24 and CL were positively correlated with RS content, whereas the proportion of DP 6–12 was negatively correlated with RS content in rice starches [10]. Additionally, the proportion of amylopectin intermediate B1 chains (DP 13–24) is the major component located in the crystalline lamellae of the rice starch granules [9,10,17]. Thus, the high proportion of DP 13–24 also contributes to an increment of crystallinity [17]. The long double helices formed by the intermediate and long chains of amylopectin in the crystalline lamellae could generate a structure with great rigidity due to the high hydrogen bonding among starch chains [15]. As a result, the stronger crystalline network formation prevents enzyme susceptibility, making the starch resistant to enzymatic hydrolysis [10,15]. An understanding of the relationship between compositional and structural characteristics and physicochemical and digestible properties of rice starches obtained from Thai commercial rice cultivars plays a crucial role in industrial application, optimization, and in enabling consumers to choose the rice genotypes according to their preferences [10,18].

Thailand possesses a large collection of elite rice cultivars with considerably high variations in starch properties [2,5,8,9,19]. From the standpoint of starchy food and non-food industries, a simple and precise classification of rice cultivars using grain appearances and starch composition and structural features is vital for the selection of rice cultivars with the proper starch functionality for rice processing industries [20,21,22]. However, the classification of Thai commercial rice cultivars based on starch chemical composition and/or structural features alone may not represent their actual physicochemical and functional properties [12,21]. Currently, starch pasting properties, which are key determinants in its functionality, have received much attention and are widely used to allow food and other biomaterial industries to select rice cultivars serving starch with desirable properties for their end-uses [18,20,21,23]. Since the pasting properties of rice starch are attributed to an interplay of many compositional and structural factors [2,8,9,10,14], the pasting properties obtained from each cultivar are intrinsically linked to its unique chemical composition and molecular structure [18,23]. However, the comprehensive information and understanding of the relationship between the variations in chemical compositions and structural features and the physicochemical and functional properties of Thai commercial rice starches with different pasting properties are still limited. Even though previous studies have pointed out the factors influencing the physicochemical and functional properties of rice flours and starches, the findings were obtained from rice varieties with slight variations in their starch pasting properties [2,8,9,24,25]. The present study, however, aims to determine the factors that may affect the starch physicochemical and functional properties of Thai rice cultivars with drastic differences in starch pasting properties. This would allow a rigorous identification of the starch compositional and structural characteristics that led to variations in starch physicochemical and functional features among tested rice cultivars.

Therefore, four rice varieties were selected based on their differences in pasting properties. Their starch chemical composition, molecular structure, physicochemical properties, and functional characteristics were characterized. The relationships between these features were then inferred to pinpoint the characteristics that putatively play an important role in defining its functionality and potential end-use applications. The obtained information would provide potential selection criteria for consumers and the industrial sectors to select suitable rice varieties, for consumption as cooked rice with sensory attributes and health benefits and for particular industrial applications, respectively.

## 2. Materials and Methods

### 2.1. Plant Materials and Growth Conditions

Seeds of the 11 Thai certified rice cultivars, namely RD6, RD21, RD29, RD37, RD41, RD43, RD57, RD61, KDML105, Phitsanulok80 and Pathumthani1, were supplied by the Pathumthani and Khon Kaen Rice Research Stations (Rice Department, Ministry of Agriculture and Cooperative, Thailand). Seeds were germinated and transplanted into 8″ plastic pots and placed in a greenhouse under natural light conditions at the Faculty of Science, Khon Kaen University from September 2019 to January 2020. A total of 4 replicates per cultivar were grown for their starch pasting property analyses. The three key starch pasting properties, including PT, PV, and SB [23], and the amylose content [19,21,26,27] of the different rice cultivars (Supplementary Table S1) were used to categorize the 11 rice cultivars into four different groups, and the representative RD57, RD29, KDML105, and RD6 cultivars were selected from each group based on their comparable photosensitivity [19] and growth duration [19] (Supplementary Table S1; highlighted in grey) for further analyses. Seeds of the four representative cultivars from each group were grown in 8” plastic pots (a plant per pot), and the grains were harvested for starch analyses. The plants were placed in a greenhouse at the Department of Biology, Faculty of Science, Khon Kaen University in the Completely Randomized Design (CRD). The environmental temperature and relative humidity were monitored and recorded at 9:00 a.m., 13:00 p.m., and 17:00 p.m. throughout the rice growth stages by Thermo-Hygrometer (Jumbo Display Wall Mount Thermo-Hygrometer, Model 13307, Deltatrak Inc., Pleasanton, CA, USA) (Supplementary Figure S1). The ranges of temperature and humidity were 16.60–40.70 °C and 20–98%, respectively, during the 2020 crop season (Supplementary Figure S1).

### 2.2. Measurements of Grain Starch and Reducing Sugar Content

Reducing sugar and total starch contents were extracted and measured according to the method of Sangwongchai et al. [2]. The reconstituted ethanol soluble fraction and the digested ethanol-insoluble pellet were used to measure the reducing sugar and starch contents using the 3,5-dinitrosalicylic acid method with d-glucose as standards [2,7].

### 2.3. Starch Isolation and Chemical Composition Analyses

*Starch isolation.* The starch was isolated from the flour of 4 representative rice cultivars via alkaline extraction as previously described [2]. The isolated starch samples were thoroughly ground, sifted through a 35 mesh (i.e., 0.50 mm) sieve (Brass Frame Stainless Mesh, Humboldt Mfg., Co., Raleigh, NC, USA), and kept at 4 °C until further analyses [2]. *Nitrogen (N) and protein content.* The N content of purified starch was estimated by using the Kjeldahl method according to the AOAC method 920.87 [28]. N values were then converted to protein content by N × 5.95. *Moisture content.* The moisture content was measured according to AACC Air Oven Method 44-19 [29] using a hot air oven (Binder Inc., Tuttlingen, Germany) at 135 °C for 2 h. *Amylose content.* Approximately 20 mg of the isolated starch was used to determine amylose content by using Amylose/Amylopectin Assay Kit (Megazyme International Ireland Ltd., Wicklow, Ireland) following the manufacturer’s recommendations.

### 2.4. Determination of Chain Length Distribution (CLD) of Amylopectin Branches

The sample preparation was performed according to a previously described method [2]. Briefly, 50 mg of each rice starch sample was dispersed in 3.8 mL of double distilled water and then boiled in a water bath at 100 °C for 1 h. The mixtures were incubated with 1 mL of the enzyme cocktail (3.6 U of pullulanase (Megazyme International Ireland Ltd., Wicklow, Ireland) and 0.5 U of isoamylase (Megazyme International Ireland Ltd., Wicklow, Ireland) in 100 mM sodium acetate buffer pH 5.0) at 40 °C for 48 h. To inactivate the enzymes, the debranched samples were augmented with 0.2 mL of 100 mM sodium hydroxide and 5 mL of deionized water, respectively. The mixtures were then centrifuged at 5000× *g* for 5 min at 25 °C and filtered through a 0.45 μm nylon filter. Finally, 1 mg/mL of the debranched amylopectin was used to analyze and determine the amylopectin CLD using a high-performance anion-exchange chromatography system equipped with a pulsed amperometric detector (HPAEC-PAD) (ICS-5000, Dionex Co., Ltd., Sunnyvale, CA, USA), according to the protocol of Lee et al. [30].

### 2.5. Determination of Starch Particle Size Distribution

The sample preparation for the determination of the starch granule size distribution via a laser diffraction particle size analyzer (Mastersizer 2000, Malvern Panalytical Co., Ltd., Worcestershire, UK) was performed following the previously described method [31]. The isolated starch of 30 mg was weighed and dispersed in 1 mL of 1% (*w*/*v*) sodium dodecyl sulfate. The dispersed starch granules were analyzed at a pump speed of 2500 rpm. The median diameter (d(0.5)), which is the granule size at which 50% of all granules by volume are smaller, was used to measure the particle size of starch granules [2].

### 2.6. Analysis of Relative Crystallinity

The degree of crystallinity of the starch granule was analyzed using Empyrean X-ray diffractometer (Malvern Panalytical Co., Ltd., Taguig, The Philippines) at 36 kV and 20 mA with a diffraction angle (2θ) of 5–40° and a scanning rate of 0.02°/s. A smooth line separating amorphous background under the X-ray diffractogram was generated using the R package cryst (https://CRAN.R-project.org/package=cryst; accessed on 7 March 2021). The area under the curve was then measured using ImageJ [32] and the % relative crystallinity was calculated as follows:

Relative crystallinity (%) = Ac/(Ac + Aa) (1)

where Ac is the crystallized area on the X-ray diffractogram, and Aa is the amorphous area on the X-ray diffractogram [33].

### 2.7. Measurements of Swelling Power and Water Solubility

The swelling power (SP) and solubility (S) of rice starch were measured according to the previously described method [2]. Firstly, rice starch powder (20 mg) was added with 1 mL double distilled water and boiled at 90 °C for 30 min. The starch slurry was then centrifuged at 1738× *g* for 30 min at room temperature. The supernatant was moved to a pre-weighted Petri dish and dried in a hot air oven (Binder, Tuttlingen, Germany) at 105 °C until the weight remained constant. The weight of the wet pellet and dried supernatant was used to calculate the SP (g/g on a dry weight basis) and S (%) values of starch granules with the following formulas [2]:

SP = [wet pellet weight × 100]/[(mass of dry starch) × (100%−% of mass of dry total soluble molecules in supernatant)](2)

S (%) = (mass of dry total soluble molecules in supernatant × 100)/mass of dry starch (3)

### 2.8. Determination of Thermal Properties

Gelatinization and retrogradation properties were determined by differential scanning calorimeter (DSC) (Diamond DSC, PerkinElmer Inc., Waltham, MA, USA), with the method modified from Tananuwong and Malila [34]. Starch slurries with a starch-to-water ratio of 1:3 (12 mg) were hermetically sealed in a DSC aluminum pan (PerkinElmer kit no. 02190062, PerkinElmer Inc., Waltham, MA, USA). The slurry was equilibrated overnight at room temperature. An empty DSC aluminum pan was used as a reference in the DSC. A temperature program was set from 30 to 90 °C at a heating rate of 10 °C/min to apply in scanning of the sample and reference DSC pans for starch gelatinization analysis. Pyris^TM^ software version 12 (PerkinElmer Inc., Waltham, MA, USA) was used to analyze the transition temperatures (i.e., the onset (To(g)), peak (Tp(g)), and conclusion (Tc(g)) temperatures), temperature range (ΔT(g)), and the enthalpy (ΔH(g)) of gelatinization from the thermograms. Thereafter, the DSC aluminum pans containing the gelatinized starch samples were kept at 4 ± 1 °C for 14 days prior to the analysis of starch retrogradation properties using the same temperature program as gelatinization analysis. The transition temperatures (i.e., the onset (To(r)), peak (Tp(r)) and conclusion (Tc(r)) temperatures), temperature range (ΔT(r)), and the enthalpy (ΔH(r)) of melting of the retrograded starch-chain structure were then analyzed. Finally, the ΔH(g) and ΔH(r) were used to calculate the degree of retrogradation (R) of gelatinized starch, following the formula [2,7].

R (%) = ΔH(r)/ΔH(g) × 100(4)

### 2.9. Determination of Pasting Properties

Pasting properties of rice starches were analyzed using a Rapid Visco-Analyser (RVA-Ezi, Newport Scientific Pty. Ltd., Warriewood, Australia) according to the AACC Method 76–21.01 [35]. Starch powder (3 g, 12% moisture basis) was mixed thoroughly with 25 g double-distilled water in the RVA aluminum canister. The starch slurries (12% *w*/*v*, 28 g of total weight) were then manually agitated for 20 s using a plastic paddle. The slurry was heated at 50 °C for 10 s with continuous stirring at 960 rpm to allow complete dispersion. The stir rotation speed was then reduced to 160 rpm, and kept for 50 s at 50 °C. Thereafter, the samples were heated to 95 °C in 3 min 42 s, and held at 95 °C for 3 min 30 s before cooling to 50 °C in 3 min 42 s and holding at 50 °C for 2 min. A constant rotating speed of the paddle (160 rpm) was applied. Differences in the 3 key RVA parameters, including pasting temperature (PT), peak viscosity (PV), and setback (SB), were recorded on the instrument. The viscosity was expressed in millipascal-seconds (mPa·s) [2,9].

### 2.10. Resistant Starch (RS) Determination

The resistant starch (RS) content was determined by the AACC Method 32-40.01 [29] according to the recommendations of the resistant starch assay kit (K-RSTAR, Megazyme International Ireland Ltd., Wicklow, Ireland). Rice starches of 100 mg were incubated with pancreatic α-amylase (40 mg, 3000 U/g, Megazyme International Ireland Ltd., Wicklow, Ireland) and amyloglucosidase (12 U, 3300 U/mL, Megazyme International Ireland Ltd., Wicklow, Ireland) in 4 mL of 100 mM sodium maleate buffer (pH 6.0) at 37 °C with continuous shaking (200 strokes/min) for 16 hr. Thereafter, the enzyme activities were inactivated by an addition of 99% (*v*/*v*) ethanol, and the sample was centrifuged at 1500× *g* for 10 min. After centrifugation, the supernatant containing the glucose released after 16 h of incubation was used to measure non-resistant starch (Non-RS) content. The pellet containing resistant starch (RS), which was starch that remained unhydrolyzed after 16 h of incubation, was dissolved and hydrolyzed by using 2M KOH and amyloglucosidase (330 U). The supernatant was then used to measure RS content. The measurements of Non-RS and RS content were performed with the glucose oxidase-peroxidase (GOPOD) reagent (Megazyme International Ireland Ltd., Wicklow, Ireland). Finally, the amounts of Non-RS and RS were calculated with the following equations:

% Non-resistant (*w*/*w*) = ΔE × F/W × 90 (5)

% Resistant Starch (*w*/*w*) = ΔE × F/W × 9.27 (6)

where:

ΔE = absorbance of sample reaction—absorbance of reagent blank

F = conversion from absorbance to micrograms calculated as 100 (μg of d-glucose)/the GOPOD absorbance for this 100 μg of d-glucose

W = the dry weight of the sample analyzed as sample weight × [(100-moisture content)/100].

### 2.11. Statistical Analysis

All parameters were measured in 3–4 biological replicates. The data analysis was performed with the SPSS^®^ statistics v.23 software (IBM Co., Ltd., Armonk, NY, USA). A one-way analysis of variance (ANOVA) and Tukey’s post hoc test were conducted for comparison of the significant differences (*p* ≤ 0.05) among the mean values. The data were expressed as mean ± standard error of the mean (SEM) [7]. Pearson correlation coefficient (r) was calculated to determine the correlation among starch compositional, structural, physicochemical, and functional parameters of the 4 rice cultivars [8].

## 3. Results

### 3.1. Screening of Rice Cultivars with Distinctive Starch Pasting Properties

The starch pasting properties and amylose content were used to categorize the 11 certified rice cultivars. The results showed that the values of the three key pasting parameters, including PT, PV, and SB, varied in the ranges of 72.70–83.98 °C, 1227.67–3447.67 mPa·s, and 270.67–2115.33 mPa·s among the 11 rice starches, respectively (Supplementary Table S1). In addition, their amylose content varied between 2.08 and 30% (Supplementary Table S1) [19,21,26,27]. The differences in the values of three RVA parameters and amylose content were concertedly used to classify the 11 Thai-certified rice cultivars into four different groups (Supplementary Table S1). We further considered their comparable photosensitivity and growth durations and selected the four suitable representative cultivars of each group as follows: (i) RD57 (high amylose content with the highest PT and SB values, and medium PV value), (ii) RD29 (high amylose content with medium PT and SB values, and the lowest PV value), (iii) KDML105 (low amylose content with low PT and SB values, and highest PV value), and (iv) RD6 (very low amylose content, the lowest PT and SB values, and medium PV value) (Supplementary Table S1; highlighted in grey).

### 3.2. Reducing Sugar and Total Starch Contents of Rice Grains

The reducing sugar and total starch content in the grains of the four Thai rice cultivars, as determined by the DNS method, were in a range of 0.65 to 0.82 mg/gFW and 713.61 to 794.86 mg/gFW, respectively (Table 1). These representative rice cultivars contained significantly different grain reducing sugar and starch contents (Table 1). The waxy rice RD6 possessed lower grain reducing sugar content (0.65 mg/gFW) than the non-waxy rice RD29 (0.82 mg/gFW) and KDML105 (0.81 mg/gFW) (Table S1 and Table 1). However, the RD6 had a higher total caryopses starch content (794.86 mg/gFW) than that of the non-waxy rice RD57 (713.61 mg/gFW) (Table 1).

### 3.3. Chemical Composition of Isolated Rice Starches

The alkaline extraction method was applied to isolate starch from the four rice flour samples, and the residual N and proteins in the isolated starch samples were analyzed (Table 1). Nitrogen and protein contents in the isolated starches of the four rice cultivars were in the ranges of 0.20–0.25% and 1.19–1.51%, respectively (Table 1). Additionally, the moisture content of the isolated starches ranged from 8.20% to 9.58% (Table 1). Previous studies reported that the amylose content of Thai commercial rice cultivars ranged from 1.69% to 30% [2,7,8,9,19]. Accordingly, the amylose content of the four rice starches were in a range of 1.65 to 23.60% in the current study (Table 1). Starch isolated from the grains of waxy rice RD6 possessed the lowest amylose content (1.65%) (Table 1). The non-waxy rice RD57 and RD29 contained higher amylose content (23.60% and 22.80%, respectively) than that of KDML105 (13.74%) (Table 1).

### 3.4. Starch Molecular Structure

#### 3.4.1. Chain-Length Distribution (CLD) of Amylopectin Branches

**Figure 1:**
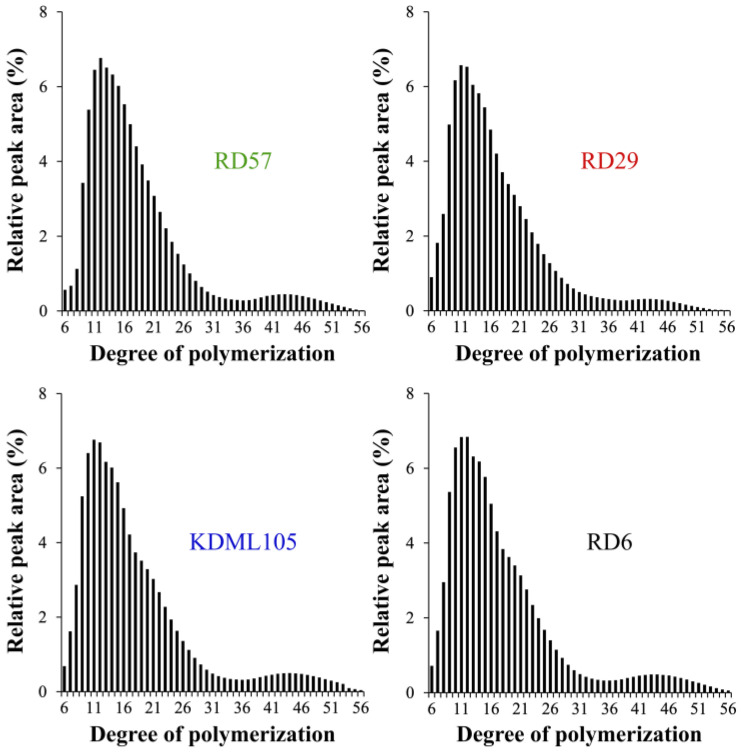
The chain length distribution (CLD) of amylopectin branches of four Thai commercial rice starches with different pasting properties.

In this study, the sensitivity of the HPAEC-PAD system was able to resolve amylopectin branch chains with DP ≤ 56. The computed peak area ratios of amylopectin branch chain types, which were categorized into A chains (DP 6–12), B1 chains (DP 13–24), B2 chains (DP 25–36), and B3+ chains (DP ≥ 37), were shown in Table 2 [2]. Among the four studied cultivars, RD57 starch possessed the smallest (27.42 ± 0.28%) and largest (57.31 ± 0.10%) amount of short A chains (DP 6–12) and B1 chains (DP 13–24), respectively, whereas the KDML105 starch had the lowest proportion (50.85 ± 0.30%) of B1 chains (DP 13–24) (Table 2). The RD29 starch contained the highest proportions (9.57 ± 0.06%) of B2 chains (DP 25–36), whereas its longer B3+ chains (DP ≥ 37) showed the lowest proportion (4.56 ± 0.33%) (Table 2). This was consistent with the shortest CL of RD29 amylopectin branches, which was 17.11 ± 0.12 AGU compared to 18.12 ± 0.12, 18.05 ± 0.22, and 17.96 ± 0.18 AGU of RD57, KDML105, and RD6 amylopectin branches, respectively (Table 2). Consistently, the normalized chromatogram of the amylopectin CLD digested from RD29 rice starch showed less B3+ chain proportion, and was obviously different from other rice starches (Figure 1).

#### 3.4.2. Starch Granule Size and Size Distribution

**Figure 2:**
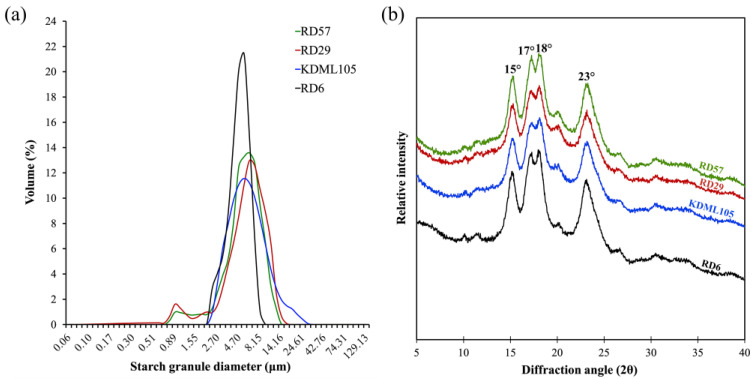
Structural characterization of starch from the caryopses of four Thai commercial rice cultivars differing in pasting properties. (**a**) Starch granule size distribution; (**b**) X-ray diffraction patterns of starch granules.

Starch granules from the four different rice cultivars showed significant variation in size, which was reported as the granule size distribution and the median particle diameter (d(0.5)) (Table 3). The results revealed that RD29 and KDML105 starches had three different types of starch granules, including A-(>15 μm), B-(5–15 μm), and C-type starch granules (<5 μm), whereas RD57 and RD6 starches only possessed A- and B-type starch granules (Table 3). In all starches, B-granules had the highest contribution to the total volume (ranged from 44.02 to 66.02%), followed by C- (ranged from 33.89 to 55.98%) and A-granules (ranged from 0.00 to 4.03%), respectively (Table 3). The volume distributions of starch granule (%) (Table 3) and the granule size distribution plot (Figure 2a) showed that KDML105 starch dominated the highest proportion (4.03 ± 0.28%) of A-granules, with a relatively low proportion of B-granules (56.74 ± 1.99), while RD6 starch occupied the highest proportion (55.98 ± 3.27%) of C- and the lowest proportion (44.02 ± 3.27%) of B-granules. Consistently, the granule size distribution plot revealed that starches from RD57 and RD29 caryopses had higher peak granule diameter (6.18 μm) than that (5.38 μm) of starches from KDML105 and RD6 rice (Figure 2a). Accordingly, the smallest (4.62 ± 0.21 μm) median particle diameters (d(0.5)) were observed in the RD6 starch (Table 3). In addition, the d(0.5) values of rice starch samples from the four rice cultivars ranged from 4.62 to 6.08 μm, which corroborated previous works [2,8] reporting the d(0.5) values of Thai rice starch granules in the range of 4.33–5.75 μm.

#### 3.4.3. Starch Granule Crystalline Structure

The relative crystallinity of starch granules can be measured by the extensively used method of X-ray diffraction [2,7,9,33]. The X-ray diffractogram of rice starches was shown in Figure 2b. The typical A-type diffraction pattern with a twin peak at diffraction angles (2θ) of 17° and 18°, and with three single peaks at 15°, 20°, and 23°, were displayed by all four rice starches (Figure 2b) [2,7]. The weak X-ray reflection peak at 2θ of 20° on the X-ray diffractogram observed in this study was a single peak of amylose–lipid complexes [2]. Additionally, the relative crystallinity of rice starches was calculated from the X-ray diffraction pattern, and the results were summarized in Table 3. The relative crystallinity of the four different rice starches ranged from 33.05 to 38.33% (Table 3), which was in agreement with the reported range of 29.9 to 45.5% for rice starches with various amylose content [10,14]. The A-type crystalline polymorph of the very low amylose RD6 starch possessed the highest crystallinity (38.33 ± 0.55%) (Table 1 and Table 3), whereas the high-amylose RD57 starch exhibited significantly higher crystallinity than that of another high-amylose RD29 starch (Table 1 and Table 3).

### 3.5. Starch Physicochemical and Functional Properties

#### 3.5.1. Swelling Power and Water Solubility

The swelling and solubility behaviors of all four rice starches were displayed in Table 3. The results showed that the swelling power and water solubility of starches from all rice grain samples were in the range of 15.75% to 39.60% and 1.47% to 27.01%, respectively (Table 3), which were similar to several previous studies [2,8,9,14]. The RD6 starch granule showed the greatest swelling power (39.60 ± 0.78%), but the lowest in water solubility (1.47 ± 0.12%) (Table 3). Moreover, starch granules isolated from KDML105 and RD6 grains swelled to a greater extent (*p* ≤ 0.05) than those of RD57 and RD29 rice grains (Table 3). Contrastingly, the water solubility of RD57 and RD29 starches was significantly (*p* ≤ 0.05) higher than that of KDML105 and RD6 starches (Table 3).

#### 3.5.2. Thermal Properties

In this study, we determined the transition temperatures, temperature range, and enthalpy during the melting of the native and retrograded starch structures to characterize the gelatinization and retrogradation behaviors of the four rice starches with different pasting properties. The results on the gelatinization behaviors, including the gelatinization temperatures (onset, To(g); peak, Tp(g); and conclusion, Tc(g)), temperature range (ΔT(g)), and enthalpy change of gelatinization (ΔH(g)) were shown in Table 4. The retrogradation behaviors, including temperatures (onset, To(r); peak, Tp(r); and conclusion, Tc(r)), temperature range (ΔT(r)), and enthalpy of melting the retrograded starch (ΔH(r)), as well as the percentage of retrogradation (R%, calculated from the percentage of ΔH(r) based on ΔH(g)), were displayed in Table 5. The differences in gelatinization and retrogradation behaviors were observed among the four rice starches (Table 4 and Table 5). For starch gelatinization properties, we found that RD57 starch showed the highest gelatinization temperatures, whereas RD29 starch showed the lowest Tp(g), Tc(g), and ΔH(g) (Table 4 and Supplementary Figure S2). Additionally, RD6 starch displayed the lowest To(g), but the highest ΔH(g) and ΔT(g) among the four cultivars (Table 4 and Supplementary Figure S2). In contrast, RD57 starch showed the lowest ΔT among the rice starch samples (Table 4 and Supplementary Figure S2). In addition, the To(g), Tp(g), Tc(g), ΔT(g), and ΔH(g) of all rice starches were in the range of 54.96–63.90 °C, 65.64–73.34 °C, 76.03–80.58 °C, 16.68–24.69 °C, and 10.74–14.28 J/g (Table 4 and Supplementary Figure S2), which is consistent with previous studies [2,8,9,14]. Regarding the starch retrogradation behaviors, the To(r), Tp(r), Tc(r), ΔT(r), ΔH(r), and %R of starch retrogradation were in the range of 38.36–42.71 °C, 54.24–56.02 °C, 64.14–71.02 °C, 21.54–32.65 °C, 2.50 to 8.07 J/g, and 17.44–68.09%, respectively, among the studied samples (Table 5 and Supplementary Figure S3). These results are consistent with previous reports on the retrogradation properties of Thai rice starches [2,8,9,14]. The results also demonstrated that RD57 starch exhibited the lowest To(r), but the highest Tp(r), Tc(r), ΔT(r), ΔH(r), and R% (Table 5 and Supplementary Figure S3). In contrast, RD6 starch displayed the lowest Tc(r), ΔT(r), ΔH(r), and R% among the rice starch samples (Table 5 and Supplementary Figure S3).

### 3.6. Resistant Starch (RS) Content

The amounts of non-resistant starch and resistant starch (RS) obtained by the AACC Method 32-40.01 were displayed in Table 6. The results revealed that RS contents of RD57, RD29, KDML105, and RD6 starches were between 0.025 and 0.153% (Table 6). The RD57 and RD29 starch samples possessed a larger amount (0.150 ± 0.01% and 0.153 ± 0.01%, respectively) of RS than that of KDML105 (0.026 ± 0.00%) and RD6 (0.025 ± 0.00%) starch samples (Table 6). Nonetheless, we found that the non-RS content did not vary among four rice starch samples, with the values of 96.80% to 98.36% (Table 6).

### 3.7. Correlation Analysis between Starch Compositional and Structural Characteristics and Starch Physicochemical and Functional Characteristics

Pearson’s correlation analysis was performed between starch compositional and structural variables, and starch physicochemical and functional variables of all rice samples, and the correlation coefficients were summarized in Table 7. Our results showed that the chemical compositions and structural features of the studied rice starches are important factors influencing their physicochemical and functional properties and RS content, as shown by several significant correlations between these two sets of parameters (Table 7) [7,9,10,14,36]. The key statistically significant correlations between starch compositional features and physicochemical properties include: (i) The amylose content negatively correlated with the SP (r = −0.967, *p* ≤ 0.01), whereas the N, protein, and amylose contents positively correlated with the water S (r = 0.706, *p* ≤ 0.05, r = 0.706, *p* ≤ 0.05, and r = 0.962, *p* ≤ 0.01, respectively) (Table 7). (ii) The N, protein, and amylose contents positively correlated with the gelatinization To(g) (r = 0.705, *p* ≤ 0.05, r = 0.705, *p* ≤ 0.05, and r = 0.707, *p* ≤ 0.01, respectively) (Table 7), which is in accordance with previous reports [8,14]. (iii) The N, protein, and amylose contents were negatively correlated with the gelatinization ∆T(g) (r = −0.755, *p* ≤ 0.01, r = −0.755, *p* ≤ 0.01, and r = −0.916, *p* ≤ 0.01, respectively) (Table 7). (iv) The reducing sugar and amylose contents were negatively correlated with the gelatinization ∆H(g) (r = −0.759, *p* ≤ 0.01, and r = −0.864, *p* ≤ 0.01, respectively) (Table 7). (v) Amylose content was positively correlated with the retrogradation Tc(r) (r = 0.842, *p* ≤ 0.01), and the N, protein, and amylose contents were positively correlated with both the retrogradation ∆T(r) (r = 0.702, *p* ≤ 0.05, r = 0.702, *p* ≤ 0.05, and r = 0.750, *p* ≤ 0.01, respectively) and the percentage of retrogradation (R%) (r = 0.706, *p* ≤ 0.05, r = 0.706, *p* ≤ 0.05, and r = 0.746, *p* ≤ 0.01, respectively) (Table 7). Lastly, (vi) amylose content was positively correlated with the RS content (r = 0.818, *p* ≤ 0.01) (Table 7).

Additionally, strong correlations were observed between amylopectin fine structure and starch physicochemical and functional features, as follows: (i) The proportion of DP 6–12 was negatively correlated with To(g) (r = −0.880, *p* ≤ 0.01), Tp(g) (r = −0.968, *p* ≤ 0.01), and Tc(g) (r = −0.710, *p* ≤ 0.01) (Table 7). (ii) The proportion of DP 25–36 was negatively correlated with To(g) (r = −0.740, *p* ≤ 0.01), Tp(g) (r = −0.936, *p* ≤ 0.01), and Tc(g) (r = −0.853, *p* ≤ 0.01) (Table 7). (iii) The proportion of DP 13–24 was positively correlated with To(g) (r = 0.942, *p* ≤ 0.01) and Tp(g) (r = 0.870, *p* ≤ 0.01), but negatively correlated with ∆T(g) (r = −0.813, *p* ≤ 0.01) (Table 7). (iv) The proportion of DP ≥ 37 and the CL were positively correlated with Tc(g) (r = 0.721, *p* ≤ 0.01 and r = 0.859, *p* ≤ 0.01, respectively) (Table 7). (v) The proportion of DP 6–12 was positively correlated with To(r) (r = 0.948, *p* ≤ 0.01), but negatively correlated with Tp(r) (r = −0.948, *p* ≤ 0.01), Tc(r) (r = −0.761, *p* ≤ 0.01), ∆T(r) (r = −0.878, *p* ≤ 0.01), ∆H(r) (r = −0.926, *p* ≤ 0.01), and R% (r = −0.883, *p* ≤ 0.01) (Table 7). (vi) The proportion of DP 13–24 was negatively correlated with To(r) (r = −0.955, *p* ≤ 0.01), but positively correlated with Tp(r) (r = 0.925, *p* ≤ 0.01), Tc(r) (r = 0.848, *p* ≤ 0.01), ∆T(r) (r = 0.933, *p* ≤ 0.01), ∆H(r) (r = 0.972, *p* ≤ 0.01), and R% (r = 0.957, *p* ≤ 0.01) (Table 7). Lastly, (vii) the proportion of DP 25–36 was positively correlated with To(r) (r = 0.794, *p* ≤ 0.01), but it showed negative correlation with Tp(r) (r = −0.827, *p* ≤ 0.01) and ∆H(r) (r = −0.761, *p* ≤ 0.01) (Table 7).

Notably, considerable correlations were revealed between the size distribution and degree of crystallinity of starch granules and their physicochemical and functional properties and RS content, as follows: (i) The proportions of C-type granule and relative crystallinity (RC.) were positively correlated with SP (r = 0.812, *p* ≤ 0.01 and r = 0.657, *p* ≤ 0.01, respectively), but negatively correlated with S (r = −0.805, *p* ≤ 0.01 and r = −0.550, *p* ≤ 0.05, respectively) (Table 7). (ii) The proportions of B-type granule and median particle size (d(0.5)) were negatively correlated with SP (r = −0.869, *p* ≤ 0.01 and r = −0.757, *p* ≤ 0.01, respectively), but positively correlated with S (r = 0.849, *p* ≤ 0.01 and r = 0.741, *p* ≤ 0.01, respectively) (Table 7). These results were also consistent with previous reports [8,9,14]. (iii) The proportion of the C-type granule was positively correlated with the gelatinization ∆H(g) (r = 0.900, *p* ≤ 0.01), whereas the proportions of the B-type granule and d(0.5) were negatively correlated with the gelatinization ∆T(g) (r = −0.732, *p* ≤ 0.01 and r = −0.589, *p* ≤ 0.05, respectively) and the ∆H(g) (r = −0.877, *p* ≤ 0.01 and r = −0.860, *p* ≤ 0.01, respectively) (Table 7). (iv) The RC was positively correlated with Tc(g) (r = 0.756, *p* ≤ 0.01) and ∆H(g) (r = 0.759, *p* ≤ 0.01) (Table 7). (v) The proportion of the C-type granule was negatively correlated with non-RS content (r = −0.782, *p* ≤ 0.01) (Table 7). (vi) The proportion of the B-type granule showed positive correlation with both non-RS content (r = 0.783, *p* ≤ 0.01) and RS content (r = 0.758, *p* ≤ 0.01) (Table 7).

## 4. Discussion

### 4.1. Variations in the Chemical Composition of the Four Thai Commercial Rice Starches with Different Pasting Properties

The reducing sugar and total starch content in the grains varied considerably among the four representative rice cultivars (Table 1). The grain reducing sugar content of waxy rice RD6 was lower than that of non-waxy rice RD29 and KDML105 (Table 1). This may be because the waxy rice RD6 accumulated relatively higher starch in the endosperm than that the non-waxy counterparts (Table 1). The difference in rice genotypes, cultivars, and starch biosynthesis could be responsible for the diversity in grain storage sugar and starch contents and starch physicochemical and functional properties [10,14]. The starches isolated from all four rice grains were also used to determine their chemical composition. The residual N and protein contents in the isolated starches did not differ among rice cultivars (Table 1). This may be because a similar alkaline starch extraction method was applied to all four rice flours, leading to no difference in N and protein contents among the tested rice starches in this study (Table 1). Additionally, the isolated starch samples were stored in the same condition before being used for further starch analyses. Therefore, a similar moisture content was observed among the four rice starches (Table 1). Previous studies reported that Thai commercial rice starches containing different amylose contents displayed significant differences in starch pasting properties [2,8,10]. Accordingly, amylose content of the four isolated starches gradually decreased from the high PT and SB, non-waxy rice RD57 and RD29 to the low PT and SB, non-waxy rice KDML105 and, finally, to the lowest PT and SB, waxy rice RD6, respectively (Table 1 and Table S1). Therefore, rice starches containing high amylose content exhibited high PT and SB values [10,14,37]. As amylose and amylose forming helical complexes with lipids can reduce the swelling potential of starch granules by holding the granule integrity during heating and shearing [37,38], starch granules possessing higher amylose content are difficult to swell and provide a higher PT value [37,38]. Hence, granules containing a higher amylose content require a higher temperature at which granule swelling, amylose leaching, and viscosity of starch suspension begins to rise during the heating process than that required for starch granules containing a lower amylose content [37,38]. During the cooling process of hot starch paste, a large amount of amylose dispersed in hot gelatinized starch can promote the formation of intra- and intermolecular hydrophobic interactions and hydrogen bonds between starch chains to form a new crystallized hard gel network rapidly, eventually resulting in an increase in SB value [8,10].

### 4.2. Differences in the Structural Features of the Four Thai Commercial Rice Starches with Different Pasting Properties

Not only starch chemical composition, but also starch molecular structure, including degree of polymerization, average chain length (CL) and chain length distribution (CLD) of amylopectin branches, amylose-lipid complexes, granular size, and degree of crystallinity, plays an important role in determining its physicochemical and functional properties and RS content [8,9,10,14]. In this study, differences in the structural features among the four different rice starches were determined. Considerable variations in starch molecular structures were observed among the four Thai rice cultivars (Table 2 and Table 3). The normalized chromatograms of the amylopectin CLD demonstrated that RD57, RD29, KDML105, and RD6 starches were A-type starches (Figure 1). The amylopectin branch chains of A-type starches typically displayed higher proportions (27.24–33.72%) of short A chains (DP 6–12) and lower proportions (4.56–7.46%) of B3+ long chains (DP ≥ 37) (Table 2) [2]. Nonetheless, the relative peak area of amylopectin long branch chains (DP ≥ 37) of RD29 appeared to be lower when compared with other cultivars (Figure 1). This is consistent with the smallest amount of B3+ long chains (DP ≥ 37) and the shortest average amylopectin branch chain length (CL) seen in this genotype (Table 2). Additionally, the highest proportions of B2 chains (DP 25–36) and the lowest proportion of B1 chains (DP 13–24) were observed in the lowest PV rice RD29 and the highest PV rice KDML105 starches, respectively (Table 2 and Table S1). On the other hand, the lowest proportion of short A chains (DP 6–12) and the highest proportion of B1 chains (DP 13–24) were seen in the medium PV rice RD57 starch (Table 2 and Table S1). These results suggested that the lowest PV of the RD29 starch could be attributed to the presences of the smallest proportion of amylopectin B3+ chains (DP ≥ 37) and the shortest CL within its relatively high amylose starch granules (Table 1 and Table 2) [8,10,14]. The maximum viscosity, or PV, is obtained when the rate of granular swelling matches the rate of granular disintegration [37]. The smallest proportion of amylopectin B3+ chains (DP ≥ 37) and the shortest CL of RD29 starch granules may not provide a strong parallel packing interaction of amylopectin double helices to maintain the integrity and stability of the swollen granules [2,10,37]. This would cause intra- and intermolecular bonds between amylopectin side chains in the RD29 starch granules to breakdown easily, leading to an increased rate of granular decomposition during heating under the excess of water and shearing force [8,9,37]. These phenomena together could be responsible for the lowest PV of RD29 starch slurry (Supplementary Table S1). Previous studies also showed that rice starches with different pasting properties typically possessed great variations in their amylopectin fine structure, granule size distribution, and degree of crystallinity [2,8,10]. Accordingly, the volume distribution of starch granules (%) and the granule size distribution plot (Table 3 and Figure 2a) showed great variation in the granule size distribution among the four rice starches with distinctive pasting properties (Figure 2a, Table 3 and Table S1). Interestingly, the waxy RD6 starch displayed the highest proportion of C- and the lowest proportion of B-granules, as well as the smallest median particle size compared to other genotypes (Table 3). Such difference could be due to the distinctive endosperm starch biosynthesis in different rice genotypes [14].

The typical A-type diffraction pattern of the rice starches consists of the strong reflections at these corresponding diffraction angles (2θ): an unresolved peak at 17° and 18° and two resolved peaks at 15° and 23°. In addition to those peaks, the X-ray diffractogram also displayed a weak single peak of amylose–lipid complexes at 2θ of 20° (Figure 2b) [2]. The pattern of the X-ray diffraction peak at 2θ of 20° demonstrated that the non-waxy rice RD57, RD29, and KDML105 starches possessed a distinctly wider base and a higher intensity of amylose–lipid peak than those of the very low amylose rice RD6 (Figure 2b). It was well-established that the non-waxy rice starches consisted of not only amylose and amylopectin but also amylose–lipid complexes [8,10,14]. Additionally, the presence of amylose molecules within starch granules not only enhanced the amylose–lipid complexes’ formation but also enhanced a defective formation of the double helix ends, which could introduce defects in the arrangements of amylopectin double helices [39,40,41]. These phenomena had a direct influence on the stability of the crystalline lamellae of the starch granules, finally resulting in a decrease in the granular crystallinity of rice starches [39,40,41]. Consequently, the relative crystallinity [42] of starches significantly varied among all cultivars with different amylose content (Table 1 and Table 3). The very low-amylose RD6 rice starch possessed the highest RC (Table 3). It was reported that the high proportion of DP 10–13 contributed to the low crystallinity [10], while the high proportion of DP 13–24 and DP > 24 could increase the thickness of crystalline lamellae within cereal starch granules, resulting in an increment of crystallinity [17]. Accordingly, we found that the RD57 and RD29 rice starches contained an equal amount of amylose, but RD29 starch possessed significantly higher amylopectin short A chains (DP 6–12) but lower intermediate B1 (DP 13−24) and long B3+ (DP ≥ 37) chains than those of RD57 starch (Table 1 and Table 2). Thus, the starch from RD29 exhibited a significantly lower crystallinity than that of RD57 (Table 1 and Table 3). Unlike previous reports [8,9,37], the equal amount of amylose in RD57 and RD29 starches did not lead to an equal rate of granular swelling during heating under excess water conditions. Due to higher crystallinity with significantly lower amounts of short A chains (DP 6–12) of amylopectin, RD57 starch granules may exhibit a slower rate of granule swelling and crystalline disruption upon heating under excess water [10,37]. RD57 starch granules consisting of amylopectin with a smaller and larger amount of short and long chains, respectively, would have larger amount of strong molecular interactions between amylopectin branch chains and their double helices to sustain the structural integrity of the swollen granule [10,37]. As a result, RD57 starch was categorized in the similar group of medium PV to RD6 starch, whereas RD29 and KDML105 rice starches were considered the lowest and highest PV starches, respectively, in this study (Supplementary Table S1).

### 4.3. Starch Compositional and Structural Characteristics in Relation to Physicochemical and Functional Properties and Resistant Starch Content

#### 4.3.1. Swelling Power and Water Solubility

Starch chemical composition and molecular structure directly affected swelling power and water solubility of starches [8,9,37]. Upon heating under excess water and minimum mechanical shear conditions, the intra-and intermolecular hydrogen bonds between starch chains were disrupted. Thereafter, water molecules would form hydrogen bonds with the free hydroxyl groups of amylose long and amylopectin branch chains [14]. These phenomena contributed to the increments of the crystalline structure disruption, with the absorption of water into the granule crystalline regions followed by amylose leaching out of the granules, resulting in an increase in granular swelling and solubility [14]. It was previously proposed that swelling is primarily a property of amylopectin, while amylose acts as an inhibitor and a promoter of swelling power (SP) and water solubility (S), respectively [2,10,37]. This agreed with our results because the high amylose rice RD57 and RD29 starches swelled significantly (*p* ≤ 0.05) less than that of the low-amylose rice KDML105 and very low-amylose rice RD6 starches, respectively (Table 1 and Table 3). Accordingly, the significantly negative correlations (r = −0.967, *p* ≤ 0.01) between amylose content and SP were observed (Table 7). Additionally, the highest SP of RD6 starch granule may be attributed to its largest proportion of C-type granules and the smallest d(0.5), which were more susceptible to heat and easier to hydrate and swell upon heating than the larger granule (Table 3) [2]. Consistently, the proportion of C-type granule was positively correlated with SP (r = 0.812, *p* ≤ 0.01), while it was negatively correlated with S (r = −0.805, *p* ≤ 0.01) (Table 7). Similarly, the proportion of B-type granule and median particle size (d(0.5)) showed negative correlation with SP (r = −0.869, *p* ≤ 0.01 and r = −0.757, *p* ≤ 0.01, respectively), but they were positively correlated with S (r = 0.849, *p* ≤ 0.01 and r = 0.741, *p* ≤ 0.01, respectively) (Table 7). Furthermore, the protein and amylose contents displayed a positive correlation with S (r = 0.706, *p* ≤ 0.05 and r = 0.962, *p* ≤ 0.01, respectively) (Table 7). The presences of amylose and soluble molecules such as protein and soluble fiber could contribute to the increase in starch solubility [8]. Notably, despite similar protein content among all four rice starches, the solubility of RD57 and RD29 starches containing high amylose content was significantly (*p* ≤ 0.05) higher than that of KDML105 and RD6 starches with low and very low amylose content, respectively (Table 1 and Table 3). Therefore, the amount of amylose is a main factor affecting the swelling power and solubility of starch granules in this study [8,10,14].

#### 4.3.2. Gelatinization Properties

The gelatinization behaviors (i.e., the gelatinization temperatures (onset, To(g); peak, Tp(g); and conclusion, Tc(g)), temperature range (ΔT(g)), and enthalpy change of gelatinization (ΔH(g))) of the four Thai rice starches with different pasting properties revealed that RD57 starch had the highest gelatinization temperatures and the lowest ∆T(g) (Table 4 and Supplementary Figure S2). This could be because RD57 starch possessed high amylose content and the smallest proportion of amylopectin short A chains (DP 6–12) (Table 1 and Table 2) [2,8,10]. Amylose molecules can form single and double helical structures and helical complexes with lipid within amorphous lamella [17]. The presences of helical and complexed molecules may retard the starch granule swelling and crystalline disruption. The gelatinization process was thus shifted to higher temperature [8,10]. A narrower range of gelatinization temperature found in RD57 samples could indicate less heterogeneity of crystallites within the starch granules. Correspondingly, amylose contents showed a positive correlation with To(g) (r = 0.707, *p* ≤ 0.01), but a significantly negative correlation with ∆T(g) (r = −0.916, *p* ≤ 0.01) (Table 7), which was in accordance with previous reports [8,43]. In addition, we observed negative correlations between the proportion of DP 6–12 and To(g) (r = −0.880, *p* ≤ 0.01), Tp(g) (r = −0.968, *p* ≤ 0.01), and Tc(g) (r = −0.710, *p* ≤ 0.01) (Table 7). It was suggested that the shorter double helices of amylopectin short chains require a lower temperature to disintegrate completely than those required for the longer double helices [8]. Thus, the presences of amylopectin short and long chains in high and low proportions, respectively, is responsible for the decrease in the gelatinization temperatures [2,8,10]. Consistently, RD29 starch containing a high proportion of DP 6–12, the lowest proportion of DP ≥ 37, the shortest CL, and also the lowest relative crystallinity showed the lowest Tp(g), Tc(g), and ∆H(g) despite its high amylose content (Table 1, Table 2, Table 3 and Table 4). We also observed that the Tc(g) was positively correlated with both the proportion of DP ≥ 37 (r = 0.721, *p* ≤ 0.01) and CL (r = 0.859, *p* ≤ 0.01), while strongly positive correlations were found between RC and Tc(g) (r = 0.756, *p* ≤ 0.01) and between RC and ∆H(g) (r = 0.759, *p* ≤ 0.01) (Table 7). Among the four rice starches, RD6 starch displayed the lowest To(g), the highest ΔT(g) and ∆H(g), which could be related to its lowest amylose content, the highest RC and the highest proportion of C-type starch granules (Table 1, Table 3 and Table 4). In case of the granule size, smaller granules, having a greater surface area to bind and absorb water molecules, normally possess efficient hydration to swell upon heating [10,44,45]. Gelatinization of RD6 starch with lowest amylose and highest proportion of the C-type granules thus initiated at the lowest temperature [10,44,45]. Moreover, the greatest RC of the RD6 samples could require the highest thermal energy for crystalline disruption, resulting in the highest ∆H(g).

#### 4.3.3. Retrogradation Properties

The retrogradation properties of the starch gels upon storage at 4 o°C for 14 days showed significant variations among the four rice starches (Table 5). Interestingly, the ΔH(r) ranged from 2.50 to 8.07 J/g, which was considerably low compared to that of starch gelatinization (Table 4 and Table 5). It was suggested that the sturdiness and the amount of the molecular ordered double helical structures established upon the retrogradation process were less than those found in the native rice starch [9]. Although the To(r) of RD57 retrograded starch sample was the lowest, its Tp(r), Tc(r), ΔT(r), ΔH(r), and R% were the highest among all retrograded starch samples (Table 5 and Supplementary Figure S3). This could be because the native RD57 starch contained large amounts of amylose and longer B3+ chains (DP ≥ 37), the largest proportion of amylopectin intermediate B1 chains (DP 13−24), and the smallest proportion of amylopectin short A chains (DP 6−12) among the four different genotypes (Table 1 and Table 2). These results are consistent with previous studies reporting that the paste of starch containing a large amount of amylose and long amylopectin branch chains was prone to form both intra- and intermolecular interactions. Those amylose and amylopectin double helices were easy to reaggregate, recrystallize, and transition into a strong retrograded starch gel network under refrigerated storage conditions [2,7,14,36]. This retrograded structure thus required high thermal energy (ΔH(r)) to melt completely [2,7,14,36]. The longer amylopectin branch chains could render the amylopectin chain segments more flexible to align and interact to form the strong double helical network within the retrograded starch [17]. Due to the complexity of the retrograded structure containing recrystallized double helices from both amylose and amylopectin molecules, heterogeneity of the crystallites was enhanced, resulting in the lowest To(r) and highest ΔT(r) of the melting endotherms. On the other hand, RD6 starch containing the smallest amount of amylose and a high proportion of amylopectin short A chains (DP 6–12) displayed the lowest Tc(r), ΔT(r), ΔH(r), and R% (Table 1, Table 2 and Table 5). Accordingly, the amylose content exhibited a significantly positive correlation with Tc(r), (∆T(r), and R% (r = 0.842, *p* ≤ 0.01, r = 0.750, *p* ≤ 0.01, and r = 0.746, *p* ≤ 0.01, respectively), and the proportion of DP 13–24 had a positive correlation with Tp(r), Tc(r), ∆T, ∆H, and R% (r = 0.925, *p* ≤ 0.01, r = 0.848, *p* ≤ 0.01, r = 0.933, *p* ≤ 0.01, r = 0.972, *p* ≤ 0.01 and, r = 0.957, *p* ≤ 0.01, respectively) (Table 7). Additionally, we found that the proportion of DP 6–12 displayed a substantially positive correlation with To(r) (r = 0.948, *p* ≤ 0.01), whereas a significantly negative correlation was observed with Tp(r) (r = −0.948, *p* ≤ 0.01), Tc(r) (r = −0.761, *p* ≤ 0.01), ∆T (r = −0.878, *p* ≤ 0.01), ∆H (r = −0.926, *p* ≤ 0.01), and R% (r = −0.883, *p* ≤ 0.01) (Table 7). Interestingly, despite the similar amylose content of the RD29 and RD57 starches, the Tp(r), Tc(r), ΔT(r), ΔH(r), and R% of the RD29 retrograded starch were lower than those of the RD57 (Table 1 and Table 5). This could be attributed to the higher proportion of DP 6–12 and the lowest proportion of DP ≥ 37 within the RD29 starch when compared to those of the RD57 starch (Table 2). The higher proportion of short A chains may render the retrograded structure of RD29 to form less ordered amylopectin crystallites [2,8,46]. Additionally, the presence of the lowest proportion of DP ≥ 37 within the RD29 starch may be responsible for a slower reaggregation and rearrangement of amylopectin double helices, as well as the formation of a weaker ordered structure than that of the RD57 retrograded starch [2,8,46]. Therefore, the RD29 retrograded starch required a lower thermal energy to dissociate completely than that required for the RD57 retrograded starch (Table 5) [2,46].

#### 4.3.4. Resistant Starch Content

In this study, the resistant starch (RS) contents of all four rice starches were in the range of 0.025 to 0.153% (Table 6), which is consistent with Chung et al. [10] who reported that rice starch has less than 1% of RS content. The RD57 and RD29 starches with large granule size and a higher amount of amylose showed a greater amount (0.150% and 0.153%, respectively) of RS than that of the large granule size KDML105 starch and the smallest granule size RD6 starch (0.027% and 0.025%, respectively), containing a smaller amount of amylose (Table 1, Table 3 and Table 6). Consistently, we found that amylose content exhibited a significantly positive correlation with the RS content (r = 0.818, *p* ≤ 0.01), and the proportion of B-type granules also showed a positive correlation with the RS content (r = 0.758, *p* ≤ 0.01) (Table 7). Starch containing higher amylose tended to have a greater amount of amylose-lipid complex. As a result, the molecular formations potentially prevent starch chains from starch digesting enzymes [10,17,47]. This could result in a higher resistance to enzyme hydrolysis of rice starch, and thus a higher RS content [10,47]. In addition, it was also shown that the ability to resist the enzymatic digestion of rice starch is attributed to its large granule size [10]. The large starch granule size could provide a small surface area, resulting in a low degree of enzymatic digestion and a large amount of RS [10]. In addition to the amylose content and starch granule size, the amylopectin chain length distribution (CLD) also had a direct effect on in vitro digestibility and RS content of native rice starch [10,37]. The longer branch chains of amylopectin molecules are prone to give higher ordered and more stable crystalline structures [2,8,13]. These molecular formations would promote the starch molecular size and the strengthening of intra- and inter-molecular hydrogen bonds between both starch chains, eventually leading to an increase in the resistance to enzymatic hydrolysis and an increase in RS content [10,17,47]. Here, the similar RS content of KDML105 and RD6 starches was observed despite their differences in amylose content and granular size (Table 1, Table 3 and Table 6). However, no differences in amylopectin fine structures were observed between KDML105 and RD6 starches (Table 2). It is, hence, suggested that the amylopectin CLD could be the important factor determining the in vitro digestion rate and RS content of these two genotypes [17].

## 5. Conclusions

Starches from the four representative Thai commercial rice cultivars with different pasting properties exhibited significant differences in their chemical compositions and molecular structures. The four rice starches possessed varied amounts of amylose, from 1.65 to 23.60%. The A-type crystalline polymorph starches with different amylopectin chain length distribution showed distinct median granule sizes ranging from 4.62 to 6.08 μm, and also differed in relative crystallinity. The diversity in the chemical composition and structural features of Thai commercial rice starches is intrinsically linked to their wide variations in swelling power, water solubility, gelatinization and retrogradation properties, and resistant starch content. As supported by the correlation coefficients, SP and ∆H increased with the declining amounts of amylose and B-type granule and the increasing amount of C-type granule. However, the opposite effects were found on S and RS content. Interestingly, the increases in the amounts of amylose and DP 13–24 and the decrease in the amount of DP 6–12 were, mainly, concertedly responsible for the increases in To(g), Tc(r), ∆T(r), ∆H(r), and R%. On the other hand, the Tp(g) and Tp(r) declined with increasing proportions of DP 6–12 and DP 25–36 and the decreasing proportion of DP 13–24, while the opposite effects were observed on To(r). Concludingly, starch physicochemical and functional properties of the four Thai rice genotypes are attributed to an interplay of many factors, such as amylose, amylopectin fine structure, granule size distribution, and crystallinity. These representative Thai rice cultivars were selected based on their distinctive pasting properties, and they also provided suitable materials for the study on the relationship between starch compositional, structural, physicochemical, and functional attributes. The results of the present study could further the understanding of the key factors affecting starch functionality, and may provide useful information for their possible industrial end uses.

## Figures and Tables

**Table 1:** The amounts of seed carbohydrates and starch chemical compositions of four Thai commercial rice cultivars differing in starch pasting properties.

Cultivars	Reducing Sugar Content (mg gFW^−1^)	Starch Content (mg gFW^−1^)	N Content (%)	Protein Content (*N* × 5.95) (%)	Amylose Content (%)	Moisture Content (%)
RD57	0.76 ± 0.02 ^ab^	713.61 ± 9.54 ^b^	0.25 ± 0.03 ^a^	1.51 ± 0.10 ^a^	23.60 ± 0.77 ^a^	9.22 ± 0.27 ^a^
RD29	0.82 ± 0.03 ^a^	761.77 ± 12.46 ^ab^	0.23 ± 0.01 ^a^	1.39 ± 0.04 ^a^	22.80 ± 1.17 ^a^	9.53 ± 0.52 ^a^
KDML105	0.81 ± 0.01 ^a^	791.43 ± 12.03 ^ab^	0.21 ± 0.03 ^a^	1.23 ± 0.10 ^a^	13.74 ± 0.69 ^b^	8.20 ± 0.34 ^a^
RD6	0.65 ± 0.05 ^b^	794.86 ± 31.56 ^a^	0.20 ± 0.01 ^a^	1.19 ± 0.03 ^a^	1.65 ± 0.04 ^c^	9.58 ± 0.65 ^a^

Values are means ± SEM. Means within each column with different superscripts are significantly different (*p* ≤ 0.05) by Tukey’s test (*n* = 4 for reducing sugar, starch, amylose, and moisture content analyses; *n* = 3 for N and protein content analyses).

**Table 2:** The amylopectin fine structure of four Thai commercial rice starches with different pasting properties.

Cultivars	Amylopectin Branch Chain Length Distribution (%)				CL (AGU)
	DP6–12 (A Chains)	DP13–24 (B1 Chains)	DP25–36 (B2 Chains)	DP ≥ 37 (B3+ Chains)	
RD57	27.42 ± 0.28 ^b^	57.31 ± 0.10 ^a^	8.73 ± 0.02 ^b^	6.54 ± 0.34 ^ab^	18.12 ± 0.12 ^a^
RD29	33.72 ± 0.29 ^a^	52.15 ± 0.09 ^b^	9.57 ± 0.06 ^a^	4.56 ± 0.33 ^b^	17.11 ± 0.12 ^b^
KDML105	32.48 ± 0.39 ^a^	50.85 ± 0.30 ^c^	9.22 ± 0.02 ^b^	7.46 ± 0.68 ^a^	18.05 ± 0.22 ^a^
RD6	32.48 ± 0.12 ^a^	51.16 ± 0.47 ^bc^	9.21 ± 0.11 ^b^	7.16 ± 0.60 ^a^	17.96 ± 0.18 ^a^

Values are means ± SEM. Means within each column with different superscripts are significantly different (*p* ≤ 0.05) by Tukey’s test (*n* = 3). Abbreviations: DP = Degree of polymerization as the number of glucose units in a branch chain length of amylopectin molecule; CL = Average chain length; and AGU: anhydroglucose unit.

**Table 3:** Granule volume distribution (%), median granule size [d(0.5)], relative crystallinity, swelling power, and water solubility of starch granules from the caryopses of four Thai commercial rice cultivars differing in starch pasting properties.

Cultivars	Volume Distributions of Starch Granule (%)			d(0.5) (μm)	Relative Crystallinity (%)	SP (g/g)	S (%)
	C-Type <5 (μm)	B-Type 5–15 (μm)	A-Type >15 (μm)				
RD57	39.88 ± 2.78 ^b^	60.12 ± 2.78 ^a^	0.00 ± 0.00 ^b^	5.59 ± 0.27 ^a^	36.76 ± 0.86 ^ab^	17.66 ± 0.76 ^c^	27.01 ± 1.15 ^a^
RD29	33.89 ± 2.05 ^b^	66.02 ± 1.98 ^a^	0.87 ± 0.07 ^b^	6.08 ± 0.10 ^a^	33.05 ± 0.74 ^c^	15.75 ± 0.55 ^c^	26.28 ± 1.60 ^a^
KDML105	39.23 ± 2.06 ^b^	56.74 ± 1.99 ^a^	4.03 ± 0.28 ^a^	5.75 ± 0.17 ^a^	35.41 ± 0.46 ^bc^	28.14 ± 0.63 ^b^	14.24 ± 0.71 ^b^
RD6	55.98 ± 3.27 ^a^	44.02 ± 3.27 ^b^	0.00 ± 0.00 ^b^	4.62 ± 0.21 ^b^	38.33 ± 0.55 ^a^	39.60 ± 0.78 ^a^	1.47 ± 0.12 ^c^

Values are means ± SEM. Means within each column with different superscripts are significantly different (*p* ≤ 0.05) by Tukey’s test (*n* = 3 for starch granule size distribution and median particle size [d(0.5)] analyses; *n* = 4 for analyses in degree of crystallinity, swelling power (SP), and solubility (S) of starch granule). Abbreviations: d(0.5) = the granule size at which 50% of all the granules by volume are smaller; SP = swelling power; and S = solubility.

**Table 4:** Gelatinization properties of starches from four Thai commercial rice cultivars with different starch pasting properties.

Cultivars	Gelatinization Properties				
	T_o(g)_ (°C)	T_p(g)_ (°C)	T_c(g)_ (°C)	∆T_(g)_ (°C)	∆H_(g)_ * (J/g)
RD57	63.90 ± 0.36 ^a^	73.34 ± 0.21 ^a^	80.58 ± 0.11 ^a^	16.68 ± 0.32 ^d^	11.89 ± 0.11 ^b^
RD29	57.28 ± 0.21 ^b^	65.64 ± 0.12 ^c^	76.03 ± 0.31 ^c^	18.75 ± 0.45 ^c^	10.74 ± 0.09 ^c^
KDML105	57.23 ± 0.24 ^b^	68.14 ± 0.15 ^b^	79.46 ± 0.13 ^b^	22.23 ± 0.20 ^b^	11.65 ± 0.29 ^b^
RD6	54.96 ± 0.15 ^c^	67.55 ± 0.39 ^b^	79.65 ± 0.26 ^b^	24.69 ± 0.33 ^a^	14.28 ± 0.12 ^a^

Values are means ± SEM. Means within each column with different superscripts are significantly different (*p* ≤ 0.05) by Tukey’s test (*n* = 4). Abbreviations: To(g) = onset temperature of gelatinization; Tp(g) = peak temperature of gelatinization; Tc(g) = conclusion temperature of gelatinization; ∆T(g) = temperature range of gelatinization (Tc(g)—To(g)); and ∆H(g) = gelatinization enthalpy. * Dry weight basis.

**Table 5:** Retrogradation properties of starches from four Thai commercial rice cultivars with different starch pasting properties.

Cultivars	Retrogradation Properties					
	T_o(r)_ (°C)	T_p(r)_ (°C)	T_c(r)_ (°C)	∆T_(r)_ (°C)	∆H_(r)_ (J/g)	R%
RD57	38.36 ± 0.15 ^b^	56.02 ± 0.11 ^a^	71.02 ± 0.16 ^a^	32.65 ± 0.31 ^a^	8.07 ± 0.11 ^a^	68.09 ± 1.64 ^a^
RD29	42.36 ± 0.11 ^a^	54.24 ± 0.11 ^b^	67.27 ± 0.12 ^b^	24.90 ± 0.17 ^b^	3.52 ± 0.10 ^b^	32.79 ± 0.56 ^b^
KDML105	42.71 ± 0.18 ^a^	54.56 ± 0.05 ^b^	67.39 ± 0.22 ^b^	24.69 ± 0.13 ^b^	3.08 ± 0.06 ^b^	26.46 ± 0.61 ^c^
RD6	42.59 ± 0.29 ^a^	54.44 ± 0.17 ^b^	64.14 ± 0.23 ^c^	21.54 ± 0.32 ^c^	2.50 ± 0.13 ^c^	17.44 ± 0.74 ^d^

Values are means ± SEM. Means within each column with different superscripts are significantly different (*p* ≤ 0.05) by Tukey’s test (*n* = 3). Abbreviations: To(r) = onset temperature of melting the retrograded starch; Tp(r) = peak temperature of melting the retrograded starch; Tc(r) = conclusion temperature of melting the retrograded starch; ∆T(r) = temperature range of melting the retrograded starch (Tc(r)—To(r)); ∆H(r) = enthalpy of melting the retrograded starch; and R% = the percentage of retrogradation.

**Table 6:** The non-resistant starch (Non-RS) and resistant starch (RS) contents of the four Thai commercial rice starches with different pasting properties.

Cultivars	Non-RS (%)	RS (%)
RD57	97.61 ± 0.51 ^a^	0.150 ± 0.01 ^a^
RD29	97.83 ± 0.87 ^a^	0.153 ± 0.01 ^a^
KDML105	98.36 ± 0.51 ^a^	0.027 ± 0.00 ^b^
RD6	96.80 ± 0.81 ^a^	0.025 ± 0.00 ^b^

Values are means ± SEM. Means within each column with different superscripts are significantly different (*p* ≤ 0.05) by Tukey’s test (*n* = 4). Abbreviations: Non-RS = non-resistant starch and RS = resistant starch.

**Table 7:** Pearson’s correlation between the compositional and structural variables and the physicochemical and functional variables of the four Thai commercial rice starches with different pasting properties.

Parameters	Sugar	Starch	NC	Protein	AC	MC	DP_6–12_	DP_13–24_	DP_25–36_	DP_≥ 37_	CL	C-Type	B-Type	A-Type	d(0.5)	RC
SP	−0.600 *	0.561 *	−0.590 *	−0.590 *	−0.967 **	−0.031	0.284	−0.577 *	0.004	0.615 *	0.415	0.812 **	−0.869 **	0.187	−0.757 **	0.657 **
S	0.551 *	−0.551 *	0.706 *	0.706 *	0.962 **	−0.040	−0.378	0.633 *	−0.112	−0.529	−0.310	−0.805 **	0.849 **	−0.128	0.741 **	−0.550 *
To(g)	0.202	−0.683 **	0.705 *	0.705 *	0.707 **	−0.026	−0.880 **	0.942 **	−0.740 **	−0.070	0.255	−0.393	0.440	−0.193	0.331	−0.007
Tp(g)	−0.075	−0.470	0.471	0.471	0.309	−0.025	−0.968 **	0.870 **	−0.936 **	0.279	0.579 *	−0.072	0.093	−0.099	0.002	0.387
Tc(g)	−0.427	−0.027	0.033	0.033	−0.284	−0.181	−0.710 **	0.395	−0.853 **	0.721 **	0.859 **	0.399	−0.447	0.193	−0.404	0.756 **
∆T(g)	−0.456	0.716 **	−0.755 **	−0.755 **	−0.916 **	−0.074	0.570	−0.813 **	0.337	0.478	0.199	0.654 *	−0.732 **	0.320	−0.589 *	0.433
∆H(g)	−0.759 **	0.279	−0.470	−0.470	−0.864 **	0.222	−0.004	−0.199	−0.239	0.454	0.384	0.900 **	−0.877 **	−0.228	−0.860 **	0.759 **
To(r)	0.127	0.593 *	−0.646 *	−0.646 *	−0.554	−0.121	0.948 **	−0.955 **	0.794 **	−0.043	−0.373	0.179	−0.260	0.387	−0.114	−0.262
Tp(r)	−0.058	−0.636 *	0.558	0.558	0.424	−0.163	−0.948 **	0.925 **	−0.827 **	0.106	0.428	−0.079	0.122	−0.209	0.006	0.369
Tc(r)	0.324	−0.538	0.692 *	0.692 *	0.842 **	−0.133	−0.761 **	0.848 **	−0.563	−0.145	0.150	−0.565	0.579 *	−0.008	0.511	−0.086
∆T(r)	0.136	−0.586 *	0.702 *	0.702 *	0.750 **	−0.025	−0.878 **	0.933 **	−0.692 *	−0.067	0.256	−0.417	0.462	−0.178	0.356	0.066
∆H(r)	0.012	−0.638 *	0.662 *	0.662 *	0.658 *	0.082	−0.926 **	0.972 **	−0.761 **	−0.039	0.297	−0.289	0.360	−0.322	0.217	0.157
R%	0.108	−0.632 *	0.706 *	0.706 *	0.746 **	0.067	−0.883 **	0.957 **	−0.689 *	−0.105	0.227	−0.390	0.458	−0.295	0.313	0.059
Non-RS	0.311	0.192	0.206	0.206	0.209	0.396	0.072	0.039	0.026	−0.214	−0.210	−0.782 **	0.783 **	0.092	0.690 *	−0.452
RS	0.315	−0.545 *	0.675 *	0.675 *	0.818 **	0.295	−0.339	0.676 *	−0.068	−0.693 *	−0.461	−0.637 *	0.758 **	−0.533	0.583 *	−0.470

Values within each column denote correlation coefficient (*r*). The stars (*) and (**) indicated statistically significant correlation at *p* ≤ 0.05 and *p* ≤ 0.01, respectively. Abbreviations: NC = nitrogen content; AC = amylose content; MC = moisture content; DP = degree of polymerization of the number of glucose units in a branch chain length of amylopectin molecule; CL = average chain length; d(0.5) = the granule size at which 50% of all the granules by volume are smaller; RC = relative crystallinity; SP = swelling power; and S = solubility; To(g) = onset temperature of gelatinization; Tp(g) = peak temperature of gelatinization; Tc(g) = conclusion temperature of gelatinization; ∆T(g) = gelatinization temperature range; ∆H(g) = gelatinization enthalpy; To(r) = onset temperature of melting the retrograded starch; Tp(r) = peak temperature of melting the retrograded starch; Tc(r) = conclusion temperature of melting the retrograded starch; ∆T(r) = temperature range of melting the retrograded starch (Tc(r)—To(r)); ∆H(r) = enthalpy of melting the retrograded starch; and R% = the percentage of retrogradation; Non-RS = non-resistant starch; and RS = resistant starch.

## Data Availability

The authors can provide the data shown in this study upon request.

## Supplementary Materials

The following supporting information can be downloaded at: https://www.mdpi.com/article/10.3390/polym15030574/s1, Figure S1: Environmental temperature and relative humidity of the cultivation location of the tested rice cultivars; Figure S2: DSC thermogram displaying endosperm starch gelatinization properties of four Thai commercial rice cultivars.; Figure S3: DSC thermogram displaying endosperm starch retrogradation properties of four Thai commercial rice cultivars; Table S1: The values of RVA parameters, amylose content, photosensitivity, and growth duration of four different groups of Thai certified rice starches.
