# Supplementary material for: Starch Chemical Composition and Molecular Structure in Relation to Physicochemical Characteristics and Resistant Starch Content of Four Thai Commercial Rice Cultivars Differing in Pasting Properties
Source: Polymers (Basel). 2023 Jan 22;15(3):574. doi: 10.3390/polym15030574 (PMC9921408; doi:10.3390/polym15030574)
Supplement: Supplementary file 1 [file polymers-15-00574-s001.zip › polymers-2101019-supplementary.pdf]

Supplementary data

Figure S1 Environmental temperature and relative humidity of the cultivation location of the tested rice cultivars.

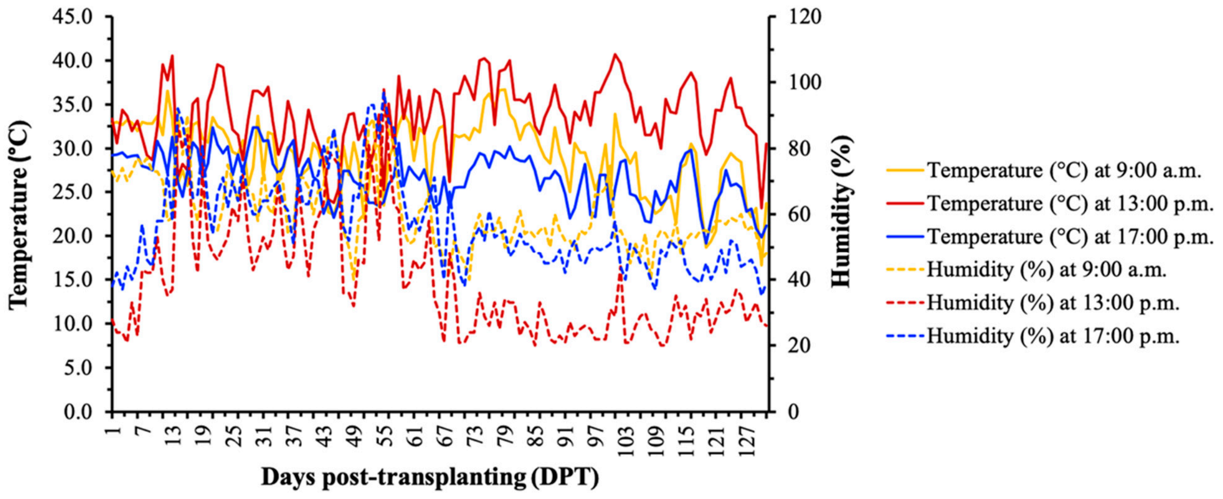

Figure S2 DSC thermogram displaying endosperm starch gelatinization properties of 4 Thai commercial rice cultivars.

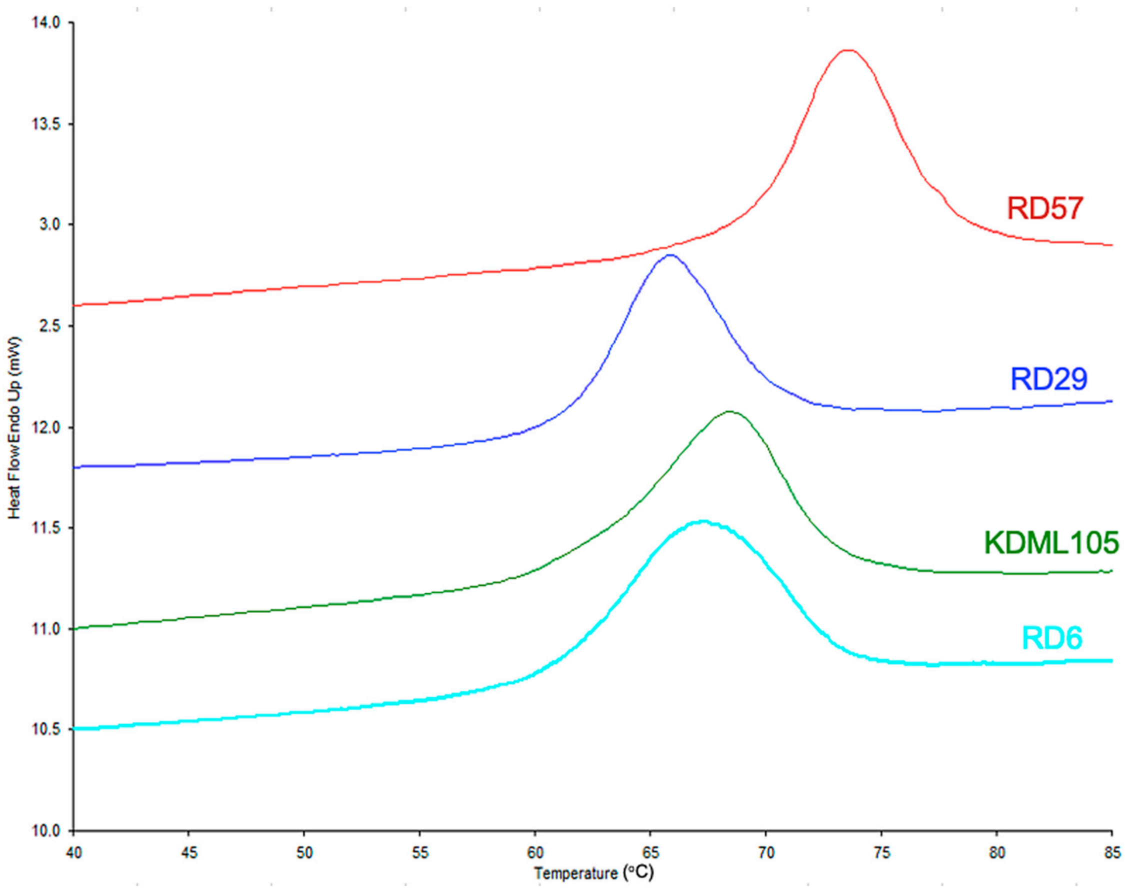

Figure S3 DSC thermogram displaying endosperm starch retrogradation properties of 4 Thai commercial rice cultivars.

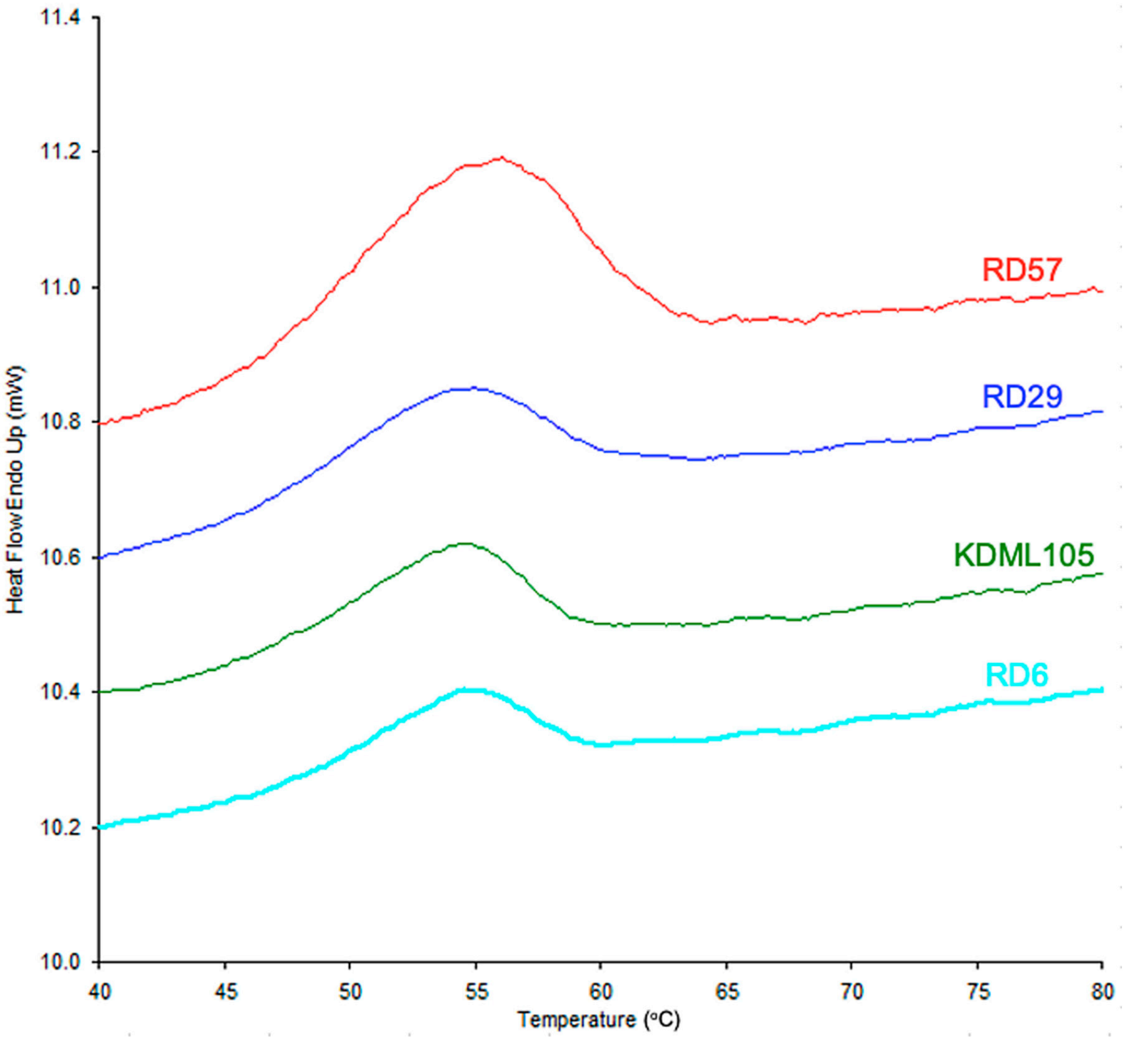

Table S1 The values of RVA parameters, amylose content, photosensitivity, and growth duration of 4 different groups of Thai certified rice starches.

Groups	Cultivars	RVA parameters			Amylose content (%)	Photosensitivity	Growth duration (Days)
		PT (°C)	PV (mPa.s)	SB (mPa.s)			
1	RD6	72.70 ± 0.45^{e}	2269.33 ± 81.06^{b}	270.67 ± 8.98^{f}	2.08	✓	110-120
	KDML105	75.67 ± 0.24^{cde}	3064.33 ± 114.62^{a}	668.67 ± 11.85^{ef}	14.38	✓	120
2	PTT1	76.47 ± 0.33^{cd}	3447.67 ± 74.38^{a}	675.67 ± 52.41^{e}	14-18.42		104-126
	RD21	75.97 ± 0.02^{cde}	3197.00 ± 178.94^{a}	950.67 ± 37.24^{de}	17-20		120-130
	PL80	73.50 ± 0.48^{de}	3073.00 ± 143.96^{a}	1159 ± 73.23^{d}	17.3	✓	120
	RD43	75.57 ± 0.73^{cde}	3016.33 ± 82.57^{a}	1183.67 ± 56.00^{d}	16-18.82		95
3	RD61	77.95 ± 0.70^{bc}	1227.67 ± 23.85^{d}	1214.00 ± 3.79^{d}	26.82		87
	RD29	76.88 ± 0.95^{cd}	1584.00 ± 40.70^{cd}	1690.33 ± 40.93^{bc}	26.6-29.4		103
4	RD37	83.98 ± 0.81^{a}	2176.33 ± 53.27^{b}	1336.67 ± 160.06^{cd}	21.6		108-118
	RD41	81.45 ± 1.28^{ab}	1642.33 ± 53.27^{cd}	1765.33 ± 167.69^{ab}	27.15-30		105
	RD57	82.37 ± 0.78^{a}	1927.67 ± 34.91^{b}	2115.33 ± 68.13^{a}	27.33		110-120

Values are means ± SEM. Means within pasting temperature (PT), peak viscosity (PV), and setback (SB) columns with different superscripts are significantly different ($p \leq 0.05$), and the means in each column indicate statistically significant difference at $p \leq 0.05$ by Tukey's test (n = 3).
